# Multi-component interventions combining psychotherapy and physical activity for children and young peoples’ mental health: A scoping review

**DOI:** 10.1371/journal.pmen.0000227

**Published:** 2025-06-16

**Authors:** Maurelle D’Sa, Nicola Relph, Shaun Liverpool, Gary Tebble, Michael Owen

**Affiliations:** Department of Social Work and Wellbeing, Faculty of Health, Social Care and Medicine, Edge Hill University, Ormskirk, Lancashire, United Kingdom; PLOS: Public Library of Science, UNITED KINGDOM OF GREAT BRITAIN AND NORTHERN IRELAND

## Abstract

Multi-component mental health interventions, combining psychotherapy and physical activity (PA), have shown potential with adult populations, but their use with children and young people (CYP) remains unclear. This scoping review aimed to synthesize existing evidence on interventions that incorporate both psychotherapy and PA for CYP (4–18 years). Using Arksey and O’Malley’s framework, and incorporating revisions from Levac, Colquhoun and O’Brien, Joanna Briggs Institute (JBI) methodology for scoping reviews, and PRISMA Extension for Scoping Reviews (PRISMA-ScR) recommendations, the review explored academic literature using five databases, as well as grey literature sources and clinical trial registries. Twenty-eight sources of evidence met the inclusion criteria and highlighted three main approaches to PA integration within the interventions: concurrent integration, asynchronous integration, and integration through PA planning and psychoeducation. A mixed pattern emerged, with six of twelve randomised controlled trials (RCTs) demonstrating statistically significant improvements in mental health and well-being outcomes. This review also raises critical questions about the development of theoretically grounded PA components and exploration of strategies for long-term sustainability and highlights the need for a structured approach to PA integration. Future research should prioritise understanding CYP’s perspectives on the feasibility and acceptability of multi-component interventions. Addressing these areas will support the development of more robust and sustainable interventions, which could become vital in future CYP mental health initiatives. Overall, this review offers novel insights into the integration of psychotherapy and PA in CYP mental health interventions and informs future research and practice in this area.

## Background

Poor mental health in children and young people (CYP) is a growing public health concern [1]. Globally, around 8–15% CYP are affected by mental health disorders [2]. Psychotherapies like cognitive behavioural therapy (CBT), mindfulness-based interventions (MBIs) and behavioural activation (BA) have shown medium to strong efficacy in improving mental health outcomes for CYP [3–7] and are therefore the most common approaches to mental health treatment worldwide. Additionally, prolonged waiting times in the UK for psychotherapy is an issue [8]. This necessitates intermediary resources and support to prevent the escalation of mental health issues [9–11].

Regular physical activity (PA) for CYP not only provides physiological benefits like improved muscular and cardiorespiratory fitness, enhanced cardiometabolic health, and reduced risk of non-communicable diseases [12], but also offers cognitive benefits such as enhanced academic performance and executive functioning, as well as improved mental health outcomes, including reduced symptoms of depression [13,14]. Additionally, PA can positively impact mental health, improving mood, boosting self-esteem, and reducing anxiety and stress [15,16]. Other advantages include improved sleep, enhanced self-perception and identity, and an overall improved quality of life [17–19]. Owing to this, PA could be considered a ‘credible adjunct treatment’ to mental health interventions [20, p. 475].

Despite existing guidelines and recommendations [21,22], PA is not widely prescribed or integrated into psychotherapy practice, which may be due to scepticism among practitioners regarding its efficacy as a treatment option [23]. Common barriers, including lack of awareness, knowledge, confidence, and training on how to promote, prescribe, and deliver PA interventions, have been identified among mental health care providers [24,25]. This highlights the necessity of developing and evaluating evidence-based approaches to effectively incorporate PA into therapeutic practice.

The synchronous combination of psychotherapy and PA has a long historical precedent, from Sigmund Freud’s walking therapy [26] and Anna Freud’s play therapy [Freud, 1928, as cited in [27], p.726] to contemporary applications including the use of mountain biking as therapy and bouldering psychotherapy [28,29]. Several studies that combine psychotherapy and PA in adults and adolescents have reported positive psychological outcomes [20,30]. However, studies often lack specificity regarding the intensity of the exercise interventions, exhibit variability in inclusion and exclusion criteria, and differ in the characteristics and interventions of their psychotherapeutic and exercise programs. Furthermore, the application of these combined interventions with CYP remains underexplored and less understood [30,31]. Given the differences in age-related psychological and developmental needs, interventions designed for adults may not be directly applicable to CYP [32–34]. Therefore, there is a critical need to expand our understanding of this area and identify gaps that could inform future research on CYP’s mental health and wellbeing.

The primary aim of this scoping review was to synthesize the types of evidence available across academic and grey literature to identify, describe, and assess multi-component mental health and psychological wellbeing interventions that include psychotherapy and PA (synchronously or asynchronously) with CYP.

## Methodology

Scoping reviews are considered an ideal tool that help determine the coverage of a topic area within literature, provide an overview of the research focus within that area, and map the available evidence [35]. A preliminary search of PsycINFO, Scopus, Prospero, and Open Science Framework was conducted and no past or ongoing synthesis of multi-component interventions that include psychotherapy and PA for mental health and psychological wellbeing improvements in CYP were identified. Taking into consideration the heterogeneous nature of existing interventions and that this area of research is not yet established, a scoping review was deemed a suitable, logical, and a needed approach. A scoping review can systematically summarise the extent, variety, and characteristics of findings on a specific topic [36], in this case making it possible to objectively determine the extent of evidence and gaps in existing literature.

This scoping review followed Arksey and O’Malley’s [37] six-stage framework for scoping review methodologies, along with revisions from Levac, Colquhoun and O’Brien [38], JBI methodology for scoping reviews [39] and PRISMA-ScR recommendations [36]. The scoping review protocol was informed by expert consultations and registered on Open Science Framework (OSF) [40] to ensure transparency and replicability.

### Stage 1: identifying the research question

The research questions for this scoping review were derived through an initial search of the existing body of literature to identify a knowledge gap and in consultation with the research team following the search. The aims of this review and the research questions were informed by consultations with experts in the field of CYP’s mental health and wellbeing. The Population, Concept, Context (PCC) framework [41] was then used to guide the formulation of the following research questions:

What are the existing multi-component mental health and psychological wellbeing interventions that include psychotherapy and PA for CYP (4 – 18 years)?

- What are the details of the interventions, (for example, content, delivery, and location)?
- What are the theories underpinning the interventions?
- What is the evidence for potential effectiveness, acceptability, and feasibility of the interventions?
- What are the facilitators and barriers to the implementation and sustainability of the interventions?

Further details and descriptions of the concepts within the research questions are presented in accordance with the PCC framework in S1 Table.

### Stage 2: identifying relevant sources of evidence

#### Search methods.

The scoping review included primary and secondary research with any study design. In addition to peer-reviewed articles published in academic journals, opinion papers, grey literature, government, council and mental health organization websites, and dissertations were also included in this scoping review.

First, an initial limited search of Scopus and PsycINFO was undertaken to identify articles on the topic. The text words contained in the titles and abstracts of relevant articles, and the index terms used to describe the articles were used to develop a full search strategy (See S1 Text) for Scopus, CINAHL, PsycINFO, MEDLINE, and Cochrane Central Register of Controlled Trials (CENTRAL). The search strategy was reviewed and guided by a chartered librarian and by experts with academic, research and practice backgrounds.

Studies included in secondary research were also checked for eligibility. This was done through pearling [42]. The primary reviewer reviewed the reference lists of all included sources of evidence to identify additional studies that may not have been identified through the literature search. Experts in the field were also contacted to signpost the primary researcher to relevant sources of information.

Grey literature sources included Open Grey, Google, Gov.UK, Gov.Wales, Northern Ireland Local Government Association and Public Health England (PHE) Publications, EThOS, ProQuest Dissertations and Thesis, NDLTD Thesis and Dissertation, Open Access Thesis and Dissertation (OATD), and the Anna Freud website. ClinicalTrials.gov, International Clinical Trials Registry Platform (ICTRP), and Google Scholar were also searched. Public health leads at London, Lancashire, Leeds, Manchester, Liverpool, Birmingham and Scottish borders council, and mental health organisations (See S2 Table) were also contacted to inquire about past or current mental health programmes that include psychotherapy and PA. All searches were completed by April 22, 2025.

#### Inclusion and exclusion criteria.

Evidence was included if it met the following criteria:

- Target population mean age ranged between 4 – 18 years and/or over 50% of the sample was within the target age range.
- Explore mental health as a primary outcome (See S1 Table for definitions).
- Included an intervention with both psychotherapeutic and PA components as defined at stage one (See S1 Table for definitions).
- Included interventions delivered worldwide, regardless of geographical location.
- Was published in English language between January 2013 and April 2025, as the study aims to review current research and trends.

Evidence was excluded if it:

- Was conducted in a psychiatric, in-patient or residential facility as this is beyond the scope of this review.
- Was published in a language other than English due to the capabilities of the review team.
- Involved CYP with physical and/or neurodevelopmental and/or neurocognitive conditions.
- Included interventions directed towards parents and practitioners.

As per the scoping review methodology framework, inclusion and exclusion criteria in this review were modified as the scoping review progressed [37,43]. Given the predominance of sources involving participants with physical and neurological conditions and a physiological or physical primary outcomes there was potential that these sources could limit the generalisability of the review findings. Such interventions are typically designed with the specific conditions of participants in mind. Consequently, the interventions are often tailored to address the unique challenges and needs associated with these conditions. For general populations, who do not share these specific profiles, the design of interventions may require significant modification to ensure their relevance and effectiveness [44]. Therefore, to maintain a clear focus on the research landscape of mental health, the decision was made to restrict the review to sources with a mental health-related primary outcome and exclude participants with physical and or neurological conditions.

### Stage 3: study selection

**Fig 1:**
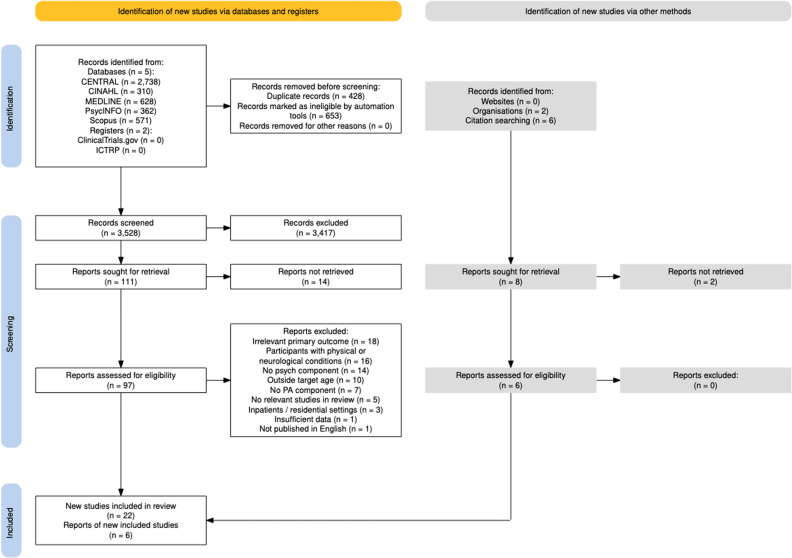
PRISMA flow diagram for the scoping review process [48].

Following the search, all identified sources were collated, and duplicates were removed using reference management (RefWorks ProQuest) and systematic review software (Rayyan [45]). Titles and abstracts, and full texts of potential sources were dual screened, manually, by two members of the research team (MO & MD) for assessment against the inclusion and exclusion criteria. The included full texts were then screened by a third reviewer (NR). Any screening disagreements were resolved through discussions within the research team (MD, NR, SL, & MO). This systematic team approach aimed to address concerns previously raised relating to rigor of scoping reviews [38]. Reasons for exclusion of sources of evidence at full text that did not meet the inclusion criteria were recorded and reported (Fig 1).

### Stage 4: charting the data

Data was extracted by the primary researcher and verified by a second reviewer (MO) using a data extraction table developed through consultation and discussion within the research team [37,38]. The data extracted included specific details about the participants, intervention setting, delivery and design, outcome measures, assessment tools, underpinning theories, study methods, and key findings relevant to the review questions.

An example of the information that guided the data extraction phase can be found in S2 Text. This list was modified and revised as necessary during the process of extracting data from each included evidence source [36]. Authors of papers were contacted to request missing or additional data, where required.

### Stage 5: collating, summarising and reporting the results

Stage 5 was divided into three steps: (i) data analysis (referred to as collating and summarising the results), (ii) reporting results, and (iii) a discussion of the results [38].

A narrative synthesis approach was used to outline the main findings and themes based on the objectives of the review. This method of data analysis was deemed suitable given its textual rather than statistical approach to analysing results and drawing conclusions [46,47].

The extracted data is presented to readers in the form of a table [36] as the scoping review aims to identify, describe, and assess mental health and psychological wellbeing multi-component interventions that include psychotherapy and PA with CYP (4 – 18 years).

A narrative summary accompanying the flow diagram and table describes how the results relate to the research question and objectives of the scoping review, the implications of the findings, and the meaning of the study results [38]. Expert consultations were used at this stage to provide content expertise and a new perspective to the findings [38].

### Stage 6: consultations

Key stakeholders (S3 Table) were consulted throughout the review process [38]. Expert consultations identified relevant literature and resources, validated key findings and commented on recommendations for future research. Expertise gained at this stage offered new perspectives and contextual meanings to the findings and overall review [38]. Outcomes from the consultations are integrated throughout the main findings and discussion sections of the review.

## Results

Searches identified 4609 sources. After duplicate removal and title and abstract screening, 111 sources were identified for full-text review. Authors (n = 27) were contacted to request missing data, with four sources sought through a librarian. Ultimately, 97 full-text sources were retrieved.

Six additional sources were identified through pearling. A final set of 28 sources of evidence describing 28 interventions met the inclusion criteria for the review (Fig 1).

### Characteristics of the reviewed sources of evidence

Study locations included Australia (n = 7), USA (n = 5), China (n = 5), UK (n = 4), Netherlands (n = 1), Denmark (n = 1), Germany (n = 1), Lebanon (n = 1), Liberia (n = 1), Sierra Leone (n = 1), and Russia (n = 1). The majority was from peer reviewed and published journal articles (n = 20). Other sources included trial registrations (n = 3), published protocols (n = 3), a book chapter (n = 1), and a chapter from a PhD dissertation (n = 1). The sources of evidence employed quantitative (n = 18), qualitative (n = 2), and mixed methods approaches (n = 7). One source of evidence was a letter to the editor outlining a quantitative study. Randomized controlled trials (RCTs) were the most prevalent study design (n = 14). The details are summarised in Table 1.

**Table 1:** Study characteristics.

Study	Design and country	Intervention	Underpinning theory	Participants	Intervention characteristics	Primary outcome and measure
Kemp et al., 2014	Programme evaluation Australia	Equine facilitated therapy (EFT)	EAGALA principles and experiential learning (PA); NI (Psyc)	n = 30 Age = 8 – 11 and 12 – 17 years Sex= Both Victims of sexual abuse and neglect and/or physical abuse Recruitment: Recruited from a facility for sexual abuse. No further information provided	Duration: average 6.6 weeks (8–11 years) and 6.4 weeks (12–17 years) (Psyc); 9–10 weeks (PA) No. of sessions: average 6.6 (8–11 years) and 6.4 (12–17 years)(Psyc) and 9–10 (PA) Frequency: Weekly (Both) Session duration: NI (Psyc); 90 minutes (PA) Facilitator: Two counsellors and four horses Setting: Clinic (Psyc)t; NI (PA) Format: Individual (Psyc), NI (PA) Mode: In person (Both)	Psychological trauma 8-11 years: CDI; CBCL 12-17 years: TSCC, BDI, BAI Upon intake and prior to in-clinic counselling (Time 1), prior to commencing EFT but after in-clinic counselling (Time 2) and upon completion of EFT (Time 3)
Frederick, Hatz and Lanning, 2015	RCT USA	Equine-assisted learning (EAL)	NI	n = 26 (I: 14) Age = 11–17 Sex = Both Meeting a minimum of one of the Texas Education Agency (2009) criteria that placed them at risk. Recruitment: Advertised through school faculty and staff, and the primary investigator.	Duration: 5 weeks No. Of sessions: NI Frequency: NI Session duration: NI Facilitator: NI Setting: NI Format: NI Mode: in person	Hope; Depression ADSHS; MDI Pre- and post-intervention as well as at four points during the intervention
Hagensen, 2015	Case study USA	Dance/movement therapy (DMT)-based holistic wellness curriculum (Holistic wellness).	DMT theory and the holistic wellness model	n = 1 Age = 11 Sex = Female Susceptible to low self-esteem Recruitment: Recruited by authors internship supervisor, NI on where recruitment took place	Duration: 6 weeks No. Of Sessions: 9 Frequency: Weekly Session duration: NI Facilitator: Researcher Setting: Private psychotherapy office Format: Individual Mode: In person	Overall wellness defined by quality of life YQOL-R; Parent surveys; Session transcriptions Pre- and post-participation (YQOL-R and Parent Surveys)
Hansen and Parker, 2015	RCT (Trial registration) Australia	Manualised integrated PA intervention, in addition to routine clinical care (psychological therapy of the clinician’s choice) (Adjunct physical activity intervention).	Behavioural activation (PA) + psychotherapeutic model of clinicians’ choice (Psyc)	n = 120 (Target) Age = 12–25 Sex = Both Depression Recruitment: Eligible participants identified through the intake and assessment process of the mental health service.	Duration: 10–12 weeks No. Of sessions: 10 Frequency: Weekly (or fortnightly) Session duration: 50 minutes Facilitator: Allied health professional Setting: Youth mental health centre Format: Individual Mode: In person	Self-report depression symptoms QIDS-SR; Qualitative interview Baseline (prior to receiving intervention), midpoint (post completing two treatment sessions but prior to completing intervention), endpoint (post intervention)
Parker et al., 2016	RCT Australia	Low-intensity interventions (problem solving therapy (PST) and PA promotion) (Physical activity intervention).	Behavioural activation (PA) + Problem-solving therapy model (Psyc)	n = 348 Age = 12–25 Sex = Both Mild mental disorder or decline in functioning Recruitment: Advertised at two participating community-based mental health centres.	Duration: 6 weeks No. Of sessions: 6 Frequency: Weekly Session duration: NI Facilitator: Research psychologists Setting: Youth mental health service centre Format: NI Mode: In person	Depression symptom severity; Anxiety symptom severity BDI-II; MADRS; BAI Baseline, post intervention
Tirlea, Truby and Haines, 2016	RCT Australia	Girls on the Go!: Interactive and experiential learning approaches including weekly themes (Health promotion programme).	Empowerment Model	n = 122 Age = 12 (mean) Sex = Female Poor body image, low self-esteem, low self-confidence, nonparticipation in sports and exercise, poor diet, or to be overweight or underweight. Recruitment: Eligible participants identified by a school welfare coordinator, nurse, counsellor, physical educator, health professional, teacher, and parent or guardian.	Duration: 10 weeks No. Of sessions: 8 Frequency: Weekly Session duration: 3 hours (introductory and follow up sessions were an hour, each) Facilitator: Youth workers + health professionals Setting: Community Format: Group Mode: In person	Self-esteem: Impairment induced by eating disorders RSES; Clinical impairment assessment Study 1 - Baseline, post intervention, 6 months follow up Study 2 - Baseline, post intervention, 6 months follow up, 9 months follow up
Frederick, 2019	Book chapter case example USA	Equine-Assisted Growth and Learning Association (Equine-assisted counselling).	EAGALA Model	n = 1 Age = 13 Sex = Female Depression Recruitment: NI	Duration: NI No. Of sessions: NI Frequency: NI Session duration: NI Facilitator: Mental health and equine professional Setting: Private facility Format: Individual Mode: In person	Depression (hope was measured at the end of the first session) ADSHS End of first session
Grasser et al., 2019	Letter to the editor USA	Dance Movement Therapy (Somatic-based interventions for addressing stress and trauma)	NI	n = 16 Age = 7 – 14 Sex = Both Refugees Recruitment: Recruited from a local resettlement agency’s Survivors of Torture programme and a local district, and were referred based on need by case managers	Duration: 12 weeks No. Of Sessions: 12 Frequency: Weekly Session duration: 90 minutes Facilitator: NI Setting: Community Format: Group Mode: In person	PTSD; anxiety symptom severity UCLA PTSD Reaction Index; SCARED Baseline and post-treatment
Marshall, Kelly and Niven, 2019	Grounded theory UK	Surf Therapy	Self Determination Theory (Identified program theory)	n = 22 Age = 8 – 23 Sex = Both Facing or at risk of mental health challenges, social and social deprivation isolation. Recruitment: Eligible young people recruited via Wave Project gatekeepers	Duration: 6 weeks No. Of sessions: 6 Frequency: Weekly Session duration: 2–3 hours Facilitator: Trained volunteers Setting: NI Format: NI Mode: In person	Participant experiences Semi structured interviews
Parker et al., 2019	RCT (Protocol) Australia	A manualized integrated PA behaviour change intervention, as per the training delivered to the clinicians, within their psychological treatment sessions (Physical activity in addition to routine clinical care).	Behaviour change techniques (PA) + psychotherapeutic model of clinicians’ choice (Psyc)	n = 960 (Target) Age = 12–25 Sex = NI Moderate or above depression levels Recruitment: Advertised at participating community-based mental health centres	Duration: 4–6 weeks No. Of sessions: 4 (anticipated) Frequency: NI Session duration: 15–20 minutes in the initial session and 5–10 minutes in subsequent sessions Facilitator: Clinicians Setting: Community Format: Individual Mode: In person	Depression symptoms QIDS-A17; Qualitative interviews Pre – and post intervention, 6 month follow up
Kliem et al., 2020	Evaluation study Germany	Klasse2000 program: A school-based health promotion program targeting nutrition, PA, self-esteem, problem-solving, critical thinking, and substance prevention through teacher-led lessons supported by expert visits and comprehensive resources (Universal health promotion, addiction, and violence prevention programme).	NI	n = 6376 (I: 2371) Age = 4^th^ Graders Sex = Both General Recruitment: School details obtained from the national office for statistics of Lower Saxony and principals contacted by postal mail	Duration: NI No. Of Sessions: 10–12 units per school year Frequency: NI Session duration: NI Facilitator: Health promoters and teachers Setting: School Format: Group Mode: In person	Wellbeing; Health-related behaviour; School and classroom atmosphere and school-based violence ILK; KINDL; Instrument to measure emotion regulation strategies in children and adolescents; SDQ-Deu; Klasse2000 evaluation by the IFT-Nord; KFN student survey; Linzer School-and classroom atmosphere Questionnaire; BVF-K
Marshall et al., 2020	Pragmatic qualitative design Liberia	Surf Therapy (Sport for development).	Aspects of Flow Theory, Plus Sport Model, Model of Sport-based Life Skills Transfer (identified program theory)	n = 23 Age = 11–25 Sex = Both From at risk communities Recruitment: Participants who had participated in surf therapy locally were invited to participate via distribution of information sheets	Duration:17 weeks No. Of sessions: 11 Frequency: NI Session duration: NI Facilitator: NI Setting: NI Format: Group Mode: In person	Participant experiences Semi structured interviews
Taranina, Starostin, and Anurov, 2020	RCT Russia	Psychotherapy and physical exercise (Physical rehabilitation)	Integrative Psychotherapeutic, Suggestive Psychotherapeutic and Cognitive behavioural therapeutic models (Psyc)	n = 90 (I: 45) Age = 11–15 Sex = Both Psychogenic depression Recruitment:NI	Duration: 8 weeks No. Of sessions: NI (Psyc); 32 (PA) Frequency: Psyc NI (Psyc); 4 x week (PA) Session duration: NI (Psyc); 1 hour (PA) Facilitator: NI Setting: NI Format: NI Mode: NI	Depression HAM-D
Bonnesen et al., 2021	RCT Denmark	Healthy High School (HHS) intervention programme: educational and environmental strategies with four main intervention components - 1) a catalogue focusing on organisational and environmental changes, 2) a teaching material, 3) a peer-led innovation workshop, and 4) a smartphone app (Wellbeing).	Socio-ecological Framework	n = 5201 Age = 16.2 (mean) Sex = Not mentioned General Recruitment: Eligible schools contacted by telephone, followed by recruitment material shared for the school manager, teachers and student counsel	Duration: 9 months No. Of Sessions: NI Frequency: NI Session duration: NI Facilitator: Teachers, student counsellors, university students, research team Setting: School and leisure Format: Group and individual Mode: In person and virtual	Wellbeing WHO-5; Cantril Ladder of Life Scale Baseline, 1st follow-up (9 months) and 2nd follow-up (20 months)
de Mooij, 2021	Micro trial Netherlands	Psychophysical intervention (adapted from the Rock and water program): Psychological topics addressed using physical exercises (Psychophysical intervention).	Cognition Theory	n = 186 (I: 60) Age = 8 – 13 Sex = Both Low scores on self-perceived competence and assertiveness Recruitment: Schools were selected from a school social work organisation’s database and invited to participate if eligible	Duration: NI No. Of sessions: 4 Frequency: NI Session duration: 1 hour Facilitator: Rock and water certified trainers Setting: School Format: Group Mode: In person	Self-worth; Self-perceived competence; Self-efficacy Dutch version of the RSES; Dutch translation of the SPPC; Dutch adaptation of the GSES Pre-test 1(five weeks before the start of the intervention), pre-test 2 (one week before the start of the intervention), Post test (one week after the intervention had ended), follow up (approximately three months after the intervention had ended).
Gehue et al., 2021	RCT Australia	‘Youth Early-intervention Study’ (‘YES’): consisting of a Social Participation (module A) and Physical Well-being (module B) (Social skills and physical wellbeing).	NI	N = 133 Age = 14 – 25 Sex = Both Clinical diagnosis of an anxiety, depressive, bipolar, or psychotic disorder according to DSM-IV Recruitment: Referred by treating psychiatrists	Duration: 16 weeks No. Of sessions: 32 Frequency: 2 x week Session duration: 2 hours Facilitator: Accredited art teacher + researcher Setting: Youth mental health clinic Format: Group Mode: In person	Functioning SOFAS Post randomization (week1, week 8, week 12, week 19), follow up (week 52)
Marshall et al., 2021	Uncontrolled mixed methods Sierra Leone	Surf Therapy (Sport for development).	Waves for Change Module of Surf Therapy	n = 58 Age = 7 – 22 Sex = Both NI Recruitment: Through local schools and community organizations	Duration: 10–12 weeks No. Of sessions: 10–12 Frequency: Weekly Session duration: 2 hours Facilitator: NI Setting: Community Format: Group Mode: In person	Wellbeing SCWS; Synthesis of evaluation reports collected from a range of stakeholders. Pre and post data (SCWS)
Moore, Woodcock and Dudley, 2021	RCT Australia	Martial arts-based intervention (Sports-based mental health intervention).	NI	n = 283 (I: 142) Age = 12 – 14 Sex = Both General Recruitment: First five eligible schools who consented after an invitation to participate were recruited. NI on how the schools were contacted	Duration: 10 weeks No. Of sessions: 10 Frequency: Weekly Session duration: 50–60 minutes Facilitator: Registered psychologist + Dan/level black-belt taekwondo instructor Setting: School Format: Group Mode: In person	Resilience CYRM Pre-intervention (baseline), post-intervention, and 12-week post- intervention (follow-up)
Shao, 2021	RCT China	Dance therapy based on Satir Model	Iceberg theory in the Satir Model (Psyc)	n = 62 (I; 32; C; 30) Mean age = 15.67 + -1.01 years (intervention) and 15.98 + -0.11 years (control) Sex = Both Depression symptoms Recruitment: Recruited by the research team from four communities	Duration: 7 weeks (psyc); 8 weeks (PA) No. Of sessions: 7 (Psyc); 8 (PA) Frequency: Weekly (Both) Session duration: 1 hour (Psyc); 2 hours (PA) Facilitator: Professional dance teacher in a university (PA); NI (Psyc) Setting: NI Format: Group Mode: NI	Mental health SCL-90; Life Satisfaction Scale; The Anxiety and Depression Subscale of Achenbach Youth Self-Report; Healthy Kids Resilience Assessment Pre and post intervention
Xu, Shen and Wang, 2021	RCT China	Aerobic exercise in combination with ACT	Exercise: NI; ACT (Psyc)	n = 83 (I:39; C: 44) Age = 12 – 19 years Sex = Both Vulnerable, symptomatic and distressed, as per dual continua model Recruitment: Eligible participants volunteered after study was advertised in schools	Duration: 6 weeks (Psyc); 8 weeks (PA) No. Of sessions: 6 (Psyc); 24 (PA) Frequency: Weekly (Psyc); 3 x week (PA) Session duration: 40–50 minutes (Psyc); 40–60 minutes (PA) Facilitator: Two post graduates in psychology as researchers and two clinical psychologists as counsellors Setting: Virtual Format: Group (except jogging) Mode: Online	Mental health GHQ-12; WEMWBS; AAQ-II Pre and post intervention
Zhang, Zhou and Zhang, 2021	RCT China	Research-based psychological counselling in combination with outdoor exercise (Research-based psychological counselling)	Research-based psychological counselling theory (Psyc)	n = 160 (I: 80) Age = 12 – 18 Sex = Both Anxiety symptoms Recruitment: Eligible participants voluntarily participated after receiving a description and explanation of the study	Duration: 8 weeks No. Of sessions: 8 (Psyc); 16 (PA) Frequency: Weekly (Psyc); 2 x week (PA) Session duration: 1 hour (Psyc); 50 minutes (PA) Facilitator: Investigator (Psyc); NI (PA) Setting: Community Format: Group Mode: In person	Psychological resilience; Depression; Anxiety; Sleep quality Healthy Kids Resilience Assessment; SDS; SAS; PSQI Pre and post intervention
Gong et al., 2022	RCT China	Narrative therapy in combination with Pilates exercise (Psychological counselling, in combination with pilates)	Narrative therapeutic model (Psychotherapy component)	n = 42 (I: 21) Age = NI Sex = NI Internet addiction Recruitment: Recruited from communities. No further information provided	Duration: 8 weeks No. Of sessions: 8 (Psyc); 16 (PA) Frequency: Weekly (Psyc); 2 x week (PA) Session duration: 45–60 minutes (Psyc); 1 hour (PA) Facilitator: NI (Psyc); Professional trainers provided Pilates training for 2 weeks prior to trial Setting: School Format: Group Mode: In person	Mental health; Status of internet addiction; Positive emotions GHQ-12; YDQ of Internet Addiction; Positive Affect Subscale in PANAS Pre and post intervention
Min and Yao, 2022	Quasi experimental design China	Positive rumination group therapy and exercise (Positive rumination-based sports prescription)	Positive rumination therapeutic model (Psyc)	n = 61 (I:29) Age = Junior high school teenagers Sex = Both Depressive symptoms Recruitment: Junior high school teenagers from four communities recruited. No further information provided	Duration: 6 weeks No. Of sessions: 6 Frequency: Weekly Session duration: 1 hour (both) Facilitator: Research team Setting: NI Format: Group Mode: In person	Depression; Anxiety; Psychological capital status BDI-II-C; DSM-5 Level 2-Anxiety-Child Age 11–17; PPQ Pre and post intervention
Moula et al., 2022	RCT UK	Dance Movement Psychotherapy (Arts therapies)	DMT theory alongside evidence from arts-based therapies and the arts for the blues model	n = 24 (I: 16) Age = 7 – 9 Sex = Both General Recruitment: Eligible participants recruited from two primary schools. No further information provided	Duration: 8 weeks No. Of Sessions: 8 Frequency: Weekly Session duration: 1 hour Facilitator: Therapist Setting: School Format: Group Mode: In person	Health-related quality of life; Areas of life functioning and wellbeing EQ-5D-Y; CORS; CSRS; SDQ with impact supplement; Semi structured interviews Pre-intervention, post-intervention, 3months follow up; 6 months follow up; 1 year follow up
Nissar, 2022	RCT (Trial registration) UK	Connect Personal, Social, Heath and Economic (PSHE) Programme: Weekly lessons focused on key wellbeing behaviours (Wellbeing programme).	ACT model	n = 520–624 (Target) Age = 7 – 11 years Sex = Both General Recruitment: Eligible participants recruited from primary schools. No further information provided	Duration: 6 months No. Of Sessions: NI Frequency: Weekly Session duration: NI Facilitator: Teachers Setting: School Format: Group Mode: In person	Wellbeing Me and My Feelings/Me and My School Questionnaire (Anna Freud Centre) Baseline and follow-up
Laker, 2023	RCT (Trial registration) UK	Psychoeducation and physical education course with PA sessions (Mental and physical health and wellbeing)	NI	n = 192 (Target) Age = 11–17 Sex = Both At risk of or suffering from low-level mental health problems Recruitment: Eligible participants recruited in school. No further information provided	Duration: 6 weeks No. Of Sessions: 6 Frequency: Weekly Session duration: 1 hour Facilitator: Local sports clubs + NHS staff Setting: Virtual Format: Group Mode: Virtual	Depression PHQ- A Baseline, six weeks, 12 weeks and six months after completion of the interventions
Luttenberger et al., 2023	Wait list-controlled trial (Protocol) Lebanon	Psychosocial bouldering intervention - YouCLIMB (Psychosocial support)	NI	n = 160 (I: 80) (Target) Age = 14–19 Sex = NI Refugees and individuals from the host community Recruitment: Recruited through schools, non-profits, social networks, social media, and outreach in informal settlements	Duration: 8 weeks No. Of sessions: 8 Frequency: Weekly Session duration: NI Facilitator: Certified climbing instructors Setting: Community Format: Group Mode: In person	Psychological wellbeing; Modes of action and outcomes of the intervention WEMWBS; Qualitative interviews. Before the intervention begins (t0, pretest) and immediately after the intervention ends (t1, post-test) (WEMWBS) Second half of the intervention (Qualitative interviews)
Prakash et al., 2024	Convergent parallel mixed methods research design - quasi-experimental single-group repeated measure design + Qualitative approach USA	Dance/movement therapy	NI	n = 13 Mean age = 10.43 years Sex = Both General population Recruitment: Flyers distributed to students in school and emailed to parents with assistance from school principals	Duration: 10 weeks No of sessions: 10 Frequency: Weekly Session duration: 50 minutes Facilitator: Author (board certified DMT and certified movement analyst) Setting: School Format: Group Mode: In person	Empathy, peer relationships, self-efficacy BES; PROMIS; PRSSF; CSES-A; weekly post-session feedback and in-depth semi-structured interviews at the end of the study Week 1, week 5 and week 10

Note: PA: physical activity; PSYC: psychotherapy; RCT: randomised controlled trial; n: sample size; MH status: mental health status; NI: no information; I: intervention group; Equine-Assisted Growth and Learning Association (EAGALA) PTSD: Post traumatic stress disorder; ACT: Acceptance Commitment Therapy.

The review included 13,361 pooled participants from 23 sources of evidence, excluding those from ongoing sources of evidence with incomplete data collection (n = 5). Participant ages ranged from 7 to 25 years, with the majority being adolescents aged 10–18 years (n = 19), followed by a mix of middle/late childhood and adolescence (n = 7). One source included participants from solely middle/late childhood age range [49] and one source of evidence mentioned school-going adolescents but did not report specific participant age ranges [50]. Most sources included both sexes (n = 21), with three focusing solely on females, and four did not specify participant sex. Overall, 22 sources implemented targeted interventions, the majority (n = 13) of which were directed towards individuals with existing mental health disorders and/or conditions. Six focused on universal interventions for CYP populations with no identified mental health condition or disorder.

### Characteristics of the reviewed interventions

#### PA integration.

The review identified three key approaches to integrating PA into psychotherapy - (i) concurrent integration, where movement is used as a vehicle for psychological change or to convey and experience the intervention’s objectives; (ii) asynchronous integration, where distinct PA sessions are delivered before or after the psychological component; and (iii) integration through PA planning and psychoeducation, where PA behaviour change strategies are incorporated into routine psychological support.

Eleven sources of evidence involved a concurrent intervention design. In most instances, the movement occurred concurrently with the psychological component of the intervention. For example, concurrent interventions included dance movement psychotherapy/therapy (DMP/DMT) that incorporated dance, movement, games, and exercises [49,51–53], surf therapy involved swimming and surfing, along with fitness and strengthening activities [54–56], and equine-assisted interventions involved equine-assisted learning and equine-assisted counselling [57,58]. Other sources that involved a concurrent intervention design described a psychosocial bouldering intervention that included climbing activities and games [59], and a psychophysical exercise intervention that used standing and balancing exercises, along with sports-based movements and exercises, that were adapted from a rock and water program [60].

The second group of interventions (n = 10) adopted asynchronous integration, that focused on sports-based activities (n = 2), mind-body exercises (n = 2), traditional fitness exercises (n = 2), dance (n = 1), mixed activities (n = 2), and equine-assisted therapy (n = 1). One of the sport-based interventions integrated martial arts exercises and pattern practice following a psychoeducation component [61], while another emphasized physical well-being and social participation, incorporating tennis and cardio-boxing alongside social activities [62]. Mind-body exercises included pilates which was conducted on a separate day from psychotherapy [63], and qigong training, although the timing of which was not reported [50]. Traditional fitness exercises were also used and included aerobic, strengthening, flexibility and joint mobility exercises. In one intervention [64] the traditional fitness exercises were conducted on a separate day from psychotherapy, while in another intervention [65] PA was adapted for home use. A dance-based intervention contained theme-based tasks [66]. One of the mixed activities intervention included a combination of activities, including yoga, PA goal setting, walking, jogging, skipping, cross country racing, badminton, and suburban leisure tourism, among others [67], while another intervention included jogging, yoga, basketball and other activities based on participant preferences [68]. The last intervention in this group combined equine facilitated therapy (EFT) with psychotherapy [69].

Finally, the third group of interventions (n = 7) that focused on integration through PA planning and psychoeducation, included PA behaviour change integrated into routine psychological support sessions delivered by the clinicians [70–72]. Interventions that integrated PA behaviour change into routine psychological support sessions were mainly manualised, and supported clinicians with integrating PA goal setting, planning, and monitoring alongside the psychological work. Additionally, interventions incorporated PA education and engagement modules within broader initiatives promoting health behaviours [73–76]. However, the intervention descriptions lacked details regarding the content of the modules and the timing of dedicated PA sessions.

#### Intervention duration and number of sessions.

Interventions lasted between 5 weeks to 8 months, with 6 weeks [50,51,54,65,71] and 8 weeks [49,59,63,64,67] being the most frequent intervention durations. Four sources did not report intervention duration [58,60,72,74]. The number of intervention sessions varied considerably, ranging from 4 to 32 sessions. Six [50,54,66,71] and eight sessions [49,59,73] were the most common durations. However, four sources did not report the number of sessions [57,58,75,76], one of these offering multiple delivery formats (workshops, lessons, app training) [75]. Five sources used different session numbers for PA and psychotherapy components [63,66–69], and one source provided data only for the PA component [64].

#### Session frequency and length.

Weekly sessions were the most common session delivery format [49–54,56,59,61,65,66,69,71,73,76], but frequencies varied across sources. Notably, seven sources did not report session frequency [55,57,58,60,72,74,75]. Three sources employed different frequencies for PA and psychotherapy components [63,67,68], and one source only provided frequency data for the PA component [64].

Session lengths for both components combined varied between 50 minutes [53,69] and 3 hours [73], with one-hour (n = 4) [49,60,61,65] and two-hour (n = 5) [50,56,62,63,67] sessions being the most common. However, data on session length was missing from nine sources [51,55,57–59,71,74–76].

The time split between the two components for concurrent interventions could not be determined due to the nature of the interventions. Out of the interventions with reported data, three asynchronous integrated interventions had the two components split equally in terms of time [50,62,65]. Other interventions dedicated one hour to psychotherapy and two hours to PA [66],one hour to psychotherapy and 50 minutes to PA [67], 40–50 minutes to psychotherapy and 40–60 minutes to PA [68], 45–60 minutes to psychotherapy and one hour to PA [63], and 10 minutes to psychoeducation as part of the psychotherapy component and the remaining 50 minutes allotted to PA [61]. Parker et al. [72] offered a 15–20-minute PA-planning intervention integrated into the first routine psychological support session which decreased to 5–10 minutes in subsequent sessions. The intervention by Hansen and Parker [70] lasted 50 minutes, however, the trial registration did not specify the time allotted to each component. Finally, each session dedicated to a health behaviour lasted 3 hours in Tirlea, Truby and Haines’ [73] intervention, with the exception of the introductory and follow up sessions, each lasting 1 hour.

#### Intervention delivery and setting.

Multidisciplinary teams were the most common mode of intervention delivery (n = 9). The teams often included volunteers, allied health professionals, exercise professionals like sports or fitness instructors as well as physiotherapists, and teachers [49,50,58,61,62,65,73–75]. Interventions were also delivered by individual professionals including clinicians, certified trainers, mental health and allied health professions [53,59,60,69–72]. Other individuals responsible for intervention delivery included trained teachers [76], trained volunteers [54], and the researcher [51]. Three sources reported implementation for only one component [63,66–68] and five sources did not contain information on who delivered the intervention [52,55–57,64].

Twelve sources reported the use of manuals, protocols, teaching material or curriculums [49,55–57,59,62,70–73,75,76] and nine sources reported on training and support provided to intervention facilitators in some form [49–54,56,62,63,68,70,72,76].

Delivery formats primarily consisted of group interventions [49,50,52,55,56,59–63,65–67,73,74,76] and two incorporated both group and individual format [68,75]. However, four sources did not provide any information on this aspect of delivery [54,57,64,71] and one source only provided information on the psychotherapy component [69].

Intervention delivery mode was predominantly in-person [49–63,67,69–74,76], with two virtual interventions [65,68] and one utilizing both modes with the use of an “app” along with sessions in-person [75]. Notably, two sources did not report the mode of delivery [64,66].

Community settings [52,56,59,67,70–73] and school settings [49,53,60,61,63,74,76] were the most common locations for intervention delivery. Of the seven school-based interventions reviewed, one was explicitly reported as being integrated into the school curriculum and delivered during regular school hours [70], while another was implemented after school hours [53]. Other settings included private clinics and offices [51,58] and a youth mental health clinic [62]. One intervention was delivered across both school and leisure settings [75]. Six sources did not report the intervention setting [50,54,55,58,64,66] and one source did not report the setting for the PA component [69].

#### Underpinning theories.

Ten sources of evidence included underpinning theories for the whole intervention. These included acceptance commitment therapy model [76], DMT theory and the holistic wellness model [51], DMT theory alongside evidence from arts-based therapies and the arts for the blues model [49], socio-ecological framework [75], cognition theory [60], waves for change model of surf therapy [56], empowerment model [73] and Equine Assisted Growth and Learning Association (EAGALA) model [58]. Two sources identified programme theories for the interventions. This included self-determination theory (SDT) [54] and aspects of flow theory, the plus sport model, and the model of sport-based life skills transfer [55].

Four sources outlined an underpinning theory for the PA components. Two out of the four interventions referenced behaviour change theories [70,71], one incorporated behaviour-change techniques including self-regulation, motivation, enjoyment, and individual tailoring without explicitly naming a theory [72], and the fourth intervention included ETF based on EAGALA principles and experiential learning [69]. The psychological components in these interventions [70–72] were underpinned by psychotherapeutic models, namely problem-solving therapy [71] while the other two were dependent on the clinicians’ choice [70,72]. The psychological component for the intervention by Kemp et al. [69] was not mentioned. Six other sources included psychotherapeutic models underpinning the psychological component [50,63,64,66–68] but did not report an underlying theory for the PA components.

#### Primary outcomes and measures.

The included sources reported a wide range of CYP mental health and psychological wellbeing outcomes, with depression being the most frequently reported primary outcome (n = 9). Two sources reported depressive symptoms as the primary outcome [70,72], while the remaining seven sources reported depression scores. Further details of the primary outcomes, specific measurement tools and timepoints can be found in Table 1 and S3 Text.

### Description of evidence findings

#### Effectiveness.

Twelve out of the sixteen RCTs were completed studies and reported effectiveness outcomes. Moula et al. [49] found a positive effect on the child outcome rating scale but no difference in health-related quality of life. Frederick, Hatz and Lanning [57] found significant effects on hope but not depression. Six studies had statistically significant improvements in mental health or psychological wellbeing [61,63,64,66–68], while Gehue et al., [62] reported improvements in functioning but did not provide information on the statistical significance. Regardless of the psychological intervention used in the four-arm study by Parker et al. [71], participants in the BA + PA group showed the greatest improvements in depression symptoms. While the intervention had a significant effect on depression scores, there was no significant difference in anxiety levels between the experimental and control groups after the intervention. Tirlea, Truby and Haines [73] noted an improvement in self-esteem but no impact on eating disorder risk. Finally, Bonnesen et al. [75] found that even though the intervention had a statistically significant effect on wellbeing, descriptive statistics showed no differences in mean and frequencies between the two student groups.

Non-RCTs also reported on study effectiveness. For example, Grasser et al. [52] reported statistically significant improvements in PTSD and anxiety symptom severity, while Hagensen [51] found positive outcomes, but statistical significance was not established due to the nature of the study. Marshall et al. [56] reported statistical significance on some well-being outcomes but inconsistent effects across sites or variables. The study by Prakash et al. [53] found no statistically significant changes in the overall aggregated scores for empathy, peer relationships, and cultural self-efficacy across participants scores. However, qualitative data revealed increase in emotional and social intelligence, strengthening of existing relationships and the development of new ones, and greater self-efficacy through multicultural awareness. Another study by Kemp et al. [69] showed EFT to be an effective therapeutic approach for CYP psychological trauma. Finally, the study by de Mooij [60] showed no improvements in self-esteem post-intervention, while Min and Yao [50] reported improvements in anxiety and psychological capital but no significant change in resilience or depression compared to controls.

#### Acceptability and feasibility.

Five sources of evidence reported on intervention feasibility, with four focusing on DMT interventions [49,51–53]. These sources [49,51–53] provided preliminary evidence supporting the potential feasibility of the DMT interventions within diverse contexts, as assessed through parent and participant surveys and analysis of sessions transcripts, participant retention rates, session attendance, or staff adherence to protocol. One study [62] also demonstrated the feasibility of a multifaceted group-based intervention targeting social participation and physical well-being. Another study [68] mentioned feasibility, however, the findings were not reported in the results section. Regarding acceptability, two sources [49,52] found that DMT interventions were acceptable to target groups, with preferences for structured sessions, the term “movement” being more appealing to boys than “dance,” and the cultural acceptance of nonverbal modalities.

### Facilitators and barriers to intervention implementation and sustainability

#### Implementation.

Ten sources of evidence identified barriers and facilitators to intervention implementation. Creating a safe, therapeutic environment was seen as crucial for open sharing in several sources of evidence [49,54–56]. Tirlea, Truby and Haines [73] emphasized the need for external facilitators and conducting interventions outside school to avoid issues like bullying, while Hagensen [51] highlighted the importance of the therapeutic relationship. Although the environmental challenges in surf therapy were noted [54], they were seen as essential for developing coping skills. Group dynamics were key facilitators in four interventions [54–56,73]. However, randomisation in grouping was a barrier to sharing personal experiences [49]. Prolonged engagement and designing engaging, structured sessions were found to facilitate positive outcomes [56,62]. A martial arts-based interventions was particularly effective in engaging clients who avoided traditional therapy [61].

Challenges to successful implementation included reduced attendance, relocation issues, and cultural concerns [56,59]. For instance, a psychosocial bouldering intervention faced difficulties in recruiting female participants due to religious or cultural norms that emphasized safeguarding girls from potentially harmful experiences outside the family environment [59]. However, adaptations like providing family-friendly groups and free transportation helped overcome barriers [52,59]. Autonomy and parental involvement were also highlighted as important factors contributing to successful implementation [51,54]. Lastly, balancing verbal and non-verbal communication was a noted challenge [49].

#### Sustainability.

Five sources of evidence addressed sustainability and post-intervention adherence. One study [61] questioned the sustainability of the martial arts-based intervention in the long run with no further discussion. Two sources [54,55] discussed the existence of opt-in clubs that provide participants with an opportunity to continue the therapy, while one study also provided participants an opportunity to offer support to new participants as mentors [55]. Another study [64] noted that to maintain the intervention’s effectiveness, ongoing exercises will be required. Finally, one study protocol [72] included plans to increase adherence as part of the clinician training for a manualised intervention to enhance sustainability.

## Discussion

This scoping review is the first to provide an overview of interventions that include both psychotherapy and PA to promote mental health and psychological well-being in CYP (4–18 years). Previous literature reviews have largely focused on adults, with limited research on youth and adolescents [20,77]. While some studies on younger populations have been included in previous reviews, they often involve participants with comorbid physical conditions and are limited to single mental health conditions. The current review identified 28 multi-component interventions that combined psychotherapy and PA and highlighted three main approaches to PA integration within the interventions: concurrent integration, asynchronous integration, and integration through PA planning and psychoeducation. Community and school settings emerged as the common locations for intervention delivery. Furthermore, interventions were most often delivered by multidisciplinary teams and were typically short-term. While the multi-component interventions in this context showed some evidence for effectiveness, the PA components were often found to lack a robust theoretical underpinning. Additionally, a significant shortcoming was the absence of models for intervention sustainability.

### What are the details of the interventions?

#### PA integration.

Based on the findings of this review, concurrent mental health interventions show promise but could face challenges in feasibility, implementation and long-term sustainability, particularly due to their resource-intensive nature, the need for specialized personnel, and access disparities based on socioeconomic factors [78–80]. For example, DMT and surf therapy interventions required trained professionals [81–83], which may limit access in regions with fewer qualified mental health providers. Additionally, further investigation into the long-term efficacy and cost-effectiveness of concurrent approaches compared to other approaches is not yet established in the current evidence-base [84,85].

While asynchronous interventions incorporating PA hold promise for improving psychological outcomes, research remains inconclusive regarding the optimal type, sequence, and timing of PA [62,86,87]. For example, the unspecified timing of PA components also complicates drawing definitive conclusions. The feasibility of asynchronous interventions in real-world settings is crucial, with home-based approaches offering potential benefits [88]. However, sustained engagement and participation are essential for the long-term success of asynchronous interventions [89]. This current review highlighted a lack of robust and consistent theoretical frameworks, which limits our understanding of how PA interventions work [90,91], hindering their potential for reusability and eventual evidence-based effectiveness [92].

Although we found positive evidence of effectiveness, the effectiveness of PA planning and psychoeducation interventions depends on clinician adherence to manualised frameworks [93], and the quality of delivery can be influenced by clinicians’ comfort with PA topics [94]. Despite promising results from interventions that incorporate PA education [73,74], the lack of detailed descriptions of the PA modules limited understanding of their efficacy. Future research is needed to identify optimal PA modalities, examining sequencing and timing, and developing evidence-based theoretical foundations, addressing the identified gaps to optimize intervention design and maximize their synergistic effects.

#### Intervention design.

Results of this review indicate that intervention settings, such as community and school environments, are crucial for CYP’s mental health interventions due to their role in child development and accessibility [19,95–101]. While both these settings offer unique advantages, challenges like school climate, resources, staff capacity and accessibility must be addressed [102–104]. Multidisciplinary teams are increasingly recognized as effective in delivering CYP’s mental health interventions [105–109]. However, the limited involvement of teachers in school-based interventions is highlighted in this review as well as the wider literature [73,110–113] and could be influenced by factors such as their perceived ability and children’s preferences, in addition to a strain on their capacity and wellbeing [113–116]. To mitigate these challenges, external mental health support teams, embedded or school-based mental health staff could be considered.

Shorter programs (6–8 weeks) were often implemented, potentially due to cost-effectiveness [117], however, the optimal treatment length remains unclear [118]. Inconsistent reporting of session details hinders comparisons between sources of evidence [119] and therefore future research is also needed to determine the optimal duration and frequency of interventions, particularly for multicomponent programs, to inform evidence-based practice.

### What are the theories underpinning the interventions?

While some sources of evidence have incorporated theories into combined interventions, many PA components lacked a clear theoretical foundation in psychology or behaviour change. This absence can hinder intervention effectiveness, engagement, and long-term sustainability [90,91,120,121]. Experts recommended the use of theoretical frameworks as theories can provide a framework for understanding behaviour change, guiding intervention design, and tailoring strategies to specific needs. For example, SDT [122] and the Model of Engagement [123] can enhance motivation and participation [13,124]. While the Transtheoretical Model (TTM) can tailor interventions to individual readiness stages [125]. Without a theoretical foundation, interventions may lack coherence, focus, and the ability to address specific barriers and facilitators to behaviour change [92,126,127]. This can then limit the ability to isolate the unique contribution of PA and predict long-term sustainability [128,129]. Integrating a relevant theory into combined interventions can enhance design, implementation, engagement, and achieve lasting positive impacts on mental health, particularly in CYP [130].

### What is the evidence for the potential effectiveness, acceptability and feasibility of the interventions?

While most interventions showed positive effects, reporting improvements in mental health and psychological wellbeing outcomes, some had mixed or null results. Additionally, six of the twelve RCTs had statistically significant findings, all of which integrated PA asynchronously. This suggests that interventions incorporating PA asynchronously may have a greater impact on mental health and well-being outcomes, potentially due to the flexibility and adaptability of such approaches [131]. However, further high-quality research, including RCT’s, is needed to confirm this relationship. Variations in outcomes may also be attributed to specific intervention components and tailoring to address individual needs. For example, the findings from Gehue et al., [62] highlight the need for interventions that address residual symptoms through the implementation of coping mechanisms and strategies to promote long-term adherence [6,64,132]. These findings emphasize the need for continued research and the development of nuanced, theoretically informed interventions to address diverse mental health concerns in CYP.

Similarly, while some sources of evidence provide preliminary evidence for the feasibility and acceptability of interventions, more research is needed to understand CYP’s perspectives on the therapeutic process and factors influencing intervention uptake and adherence including preferences for intervention structure, content, and delivery mode [133].

### What are the facilitators and barriers to the implementation and sustainability of the interventions?

This review also highlights the importance of the therapeutic relationship, social interaction, resources, and environmental factors in facilitating combined psychotherapy and PA interventions for CYP. While these factors align with broader literature [134–137], challenges such as individual needs within group settings and limited resources require addressing [138]. A theoretical model and government-level policies could enhance intervention implementation by providing practitioners with tools, strategies, and systemic changes to address these challenges [139]. By integrating these considerations, practitioners and researchers can design more effective interventions that foster positive implementation experiences and long-term behaviour change to enhance sustainability.

## Recommendations

The aim of this review was to highlight gaps in current knowledge and establish future research priorities. Therefore, based on the findings of the review, the following research recommendations emerge. First, the review indicates that while interventions with asynchronous PA integration show promise for improving psychological outcomes, further, high-quality research is needed to establish optimal PA modalities and enhance intervention design. The question regarding number of sessions, which could vary based on CYP needs, developmental stage, and capability [140,141], also remains underexplored, indicating a need for further research in the area. Additionally, the consultation process revealed that this approach is becoming increasingly common in practice; however, it is not adequately reflected in the literature. Therefore, it is recommended that future research efforts focus on documenting and analysing these emerging practices to bridge the gap between real-world application and academic understanding [142,143].

Secondly, consistent reporting of intervention characteristics is essential for better comparisons and conclusions. In the future, researchers could adopt standardised outcome measures to improve cross-study comparability and ensure clarity in defining mental health constructs [144].

Moreover, given that the PA components often lacked an underpinning theoretical framework, integrating and testing psychological and behaviour change theories within PA components could enhance sustainability and real-world applicability of future interventions [121]. Future research could then explore the long-term sustainability of interventions, examining factors that influence continued engagement and outcomes over time. Investigating behaviour change mechanisms, along with the use of long-term follow-up assessments, may also inform the development of more effective interventions for CYP mental health and assess adherence to coping strategies [130].

The current review also identified the importance of establishing therapeutic relationships as an important facilitator for intervention delivery. Future research could explore the processes involved in developing robust therapeutic relationships across diverse settings. Additionally, it would be valuable to examine the interplay between the nature of therapeutic relationships and the type of PA incorporated into interventions. Understanding how different approaches impact therapeutic relationships could provide important insights into optimising intervention efficacy [144,145].

Finally, the review identified a lack of research relating to the feasibility and acceptability of interventions with CYP and practitioners in this area. Therefore, feasibility and acceptability research could prioritise participant as well as practitioner perspectives and preferences on intervention structure, content, and delivery mode. Examining feasibility and acceptability from the perspective of practitioners is essential, as their role is critical in reaching and engaging youth, and their insights can provide valuable information on real-world implementation challenges and opportunities [146]. Additionally, the UK Medical Research Council’s (MRC) guidelines for developing and evaluating complex interventions emphasise the importance of evaluating both the feasibility and acceptability of interventions to inform decisions regarding progression to the next phase of assessment [129]. Investigating how to individualise interventions in line with the unique needs of CYP could improve engagement and outcomes [13]. The personalised nature of therapy for CYP should be reflected in the design of interventions, with careful attention to duration, intensity, and modality to ensure they align with the therapeutic and developmental needs of participants. Additionally, the involvement of stakeholders, including CYP, teachers and parents, could enhance the intervention development and implementation process, and further support intervention success [129,147–149].

Additionally, the scoping review identified some implications for practice.

The predominance of short-term interventions reviewed underscored the necessity of efficient resource utilization within the overburdened systems. Prioritising multidisciplinary teams could enhance the delivery of comprehensive and cost-effective interventions [107,150,151]. For example, schools could better leverage community teams, external mental health support teams, embedded or school-based mental health staff, like the Educational Mental Health Professionals (EMHPs) to bolster their resources, creating a multi-professional team, with diverse professional perspectives, without overwhelming educators. Additionally, the involvement of multi-professional teams adds another layer of safety and a robust professional framework, ensuring comprehensive support and collaboration in the intervention process [152]. A collaborative approach could also be emphasized to promote shared ownership and enhance the effectiveness of interventions [153,154]. It could also ensure that interventions are responsive to the specific needs and preferences of CYP and their broader support network. This model not only improves the relevance of interventions but also fosters stronger buy-in from participants, thereby contributing to their overall success and sustainability [155].

The current review also identified a lack of planning in relation to long-term health behaviour change within interventions. Therefore, future intervention design could consider the readiness and motivational needs of CYP, as emphasized by the TTM and SDT. Assessing CYP for intervention suitability and appropriateness, alongside evaluating their presenting mental health condition, could be essential for tailoring the intervention effectively to meet their specific needs. Personalizing or tailoring interventions to address the specific needs or issues of individual CYP enhances the effectiveness and sustainability of interventions in real-world settings [156,157]. Although more resource-intensive upfront, tailoring strategies to align with community values and context can enhance both initial uptake and long-term sustainability [158].

Lastly, several facilitators and barriers to effective intervention delivery were identified in the current review. Fostering social interaction and group cohesion enhances participant engagement and support [159,160]. Therefore, practitioners should prioritise establishing strong therapeutic relationships and safe environments to facilitate effective intervention implementation and participant engagement.

However, it is important to note that the ability of scoping reviews to offer clear clinical or policy recommendations may be restricted [35], and the practical recommendations should therefore be approached with caution.

## Strengths and limitations

This scoping review offers an original contribution to the field of CYP’s mental health and psychological wellbeing, based on relevant and current research, following established frameworks [36–39] to ensure a rigorous and transparent approach. A priori protocol [40], inclusion of a chartered librarian, expert consultations, and dual-reviewer assessments also strengthened the process and mitigated potential biases. This strengthened the review’s reliability and addressed concerns relating to the rigor of scoping reviews [161].

While scoping reviews are effective in mapping out the existing literature, they do not typically involve a critical appraisal of the quality of the included sources of evidence [162]. This lack of quality assessment may impact the overall robustness of the findings and limited ability to draw definitive conclusions regarding the effectiveness or reliability of the evidence [41]. Additionally, the heterogeneity of the included sources of evidence hindered the ability to draw definitive conclusions across the sources. However, a notable strength of this review was its ability to synthesise the diverse interventions into three distinct categories, a novel approach that provides a valuable foundation for future research.

## Conclusion

In conclusion, the current body of evidence suggests that combining PA and psychotherapy hold promise for mental health outcomes in CYP. While the use of multidisciplinary teams to deliver such interventions is promising, the current evidence base is insufficient to definitively advocate for this approach. A critical question arising from this review pertains to the ideal number of sessions and reporting of intervention characteristics. Furthermore, the field may benefit from a more structured approach, potentially informed by a theoretical or model-driven framework, to guide intervention design, implementation, and evaluation. Ongoing research to further understand CYP’s perspectives on feasibility and acceptability of combining PA and psychotherapy is also essential.

By addressing these areas, researchers can contribute to the development of more robust and sustainable interventions for improving CYP mental health and wellbeing. The combined intervention has the potential to become a cornerstone resource in CYP mental health initiatives.

## Supporting information

S1 Table Population, concept, context (PCC) framework for determining the eligibility of the research question. (DOCX)

S2 Table Mental health organisations. (DOCX)

S3 Table Expert involvement. (DOCX)

S1 Text Search strategy. (DOCX)

S2 Text Data extraction guidance. (DOCX)

S3 Text Primary outcome measures. (DOCX)

S4 Text Search history (CINAHL complete). (PDF)

S1 Checklist PRISMA-ScR checklist. (DOCX)
