# Supplementary material for: Multi-component interventions combining psychotherapy and physical activity for children and young peoples’ mental health: A scoping review
Source: PLOS Ment Health. 2025 Jun 16;2(6):e0000227. doi: 10.1371/journal.pmen.0000227 (PMC12798439; doi:10.1371/journal.pmen.0000227)
Supplement: S1 Table — (DOCX) [file pmen.0000227.s001.docx]

# **S1 Table. Population, concept, context (PCC) framework for determining the eligibility of the research question**

| Population | School aged CYP (4 – 18) years |
| --- | --- |
| Concept | Intervention: including both psychotherapy ‘and’ PA components in any form (for example, implemented synchronously or asynchronously). For the purpose of this review, the term ‘multi-component’ will be used to describe interventions that include both components of psychotherapy and PA, unless otherwise specified (for example, the primary authors use of another term, like adjunct, concurrent, or integrated, in studies being reviewed).  Psychotherapy, defined as “any psychological service provided by a trained professional that primarily uses forms of communication and interaction to assess, diagnose, and treat dysfunctional emotional reactions, ways of thinking, and behaviour patterns” (American Psychological Association, n.d.)  Physical activity (PA), defined as ‘any bodily movement produced by skeletal muscles that requires energy expenditure’ (World Health Organization, 2022). This includes exercise, i.e., ‘a subcategory of physical activity that is planned, structured, repetitive, and purposive, in the sense that the improvement or maintenance of one or more components of physical fitness is the objective’ (Centers for Disease Control and Prevention (CDC), 2017, cited in Dasso, 2018, p. 46).  Intervention ‘delivery’: includes all aspects of intervention delivery including but not limited to the form (or order) of the psychotherapy and PA components delivered, the individual(s) delivering the intervention, dosage, timing, and mode of delivery.  Outcomes:  Mental health is operationally defined using the dual continua model of mental health (Keyes, 2002, 2005) where it is viewed as a complete state including positive mental health and not just an absence of mental illness (Westerhof and Keyes, 2010).  Positive mental health has been defined as ‘the experience of positive feelings or subjective wellbeing and functioning fully or optimally (Huppert, 2005), encompassing individual resources such as life satisfaction (Diener,1984), positive emotions (Fredrickson, 2001), meaning and purpose in life (Steger et al., 2006), resilience (Bonanno, 2004), character strengths (Peterson and Seligman, 2004), and interpersonal relationships (Reisand Gable, 2003)’ (cited in Iasiello and Van Agteren, 2020, p. 2).  Mental illness (as called mental disorder or disease) is defined by the APA as ‘any condition characterised by cognitive and emotional disturbances, abnormal behaviours, impaired functioning, or any combination of these; that cannot be accounted for solely by environmental circumstances and may involve physiological, genetic, chemical, social, and other factors’ (American Psychological Association, n.d.).  Psychological wellbeing is operationally defined as a state of complete mental health (i.e., high positive mental health without a mental illness or diagnosis.  *Several terminologies exist in the area of mental health and mental illness. The scoping review will include mental health / illness / disorder / psychological wellbeing as described or defined by the primary author of included articles. |
| Context | Studies published since January 2013 will be included since the study aims to review current trends and research on mental health and psychological wellbeing interventions within the last 10 years.  Interventions delivered across multiple settings will be included, except for those conducted in psychiatric facilities and inpatient mental health settings owing to differences with the target population. |
