# Supplementary material for: Multi-component interventions combining psychotherapy and physical activity for children and young peoples’ mental health: A scoping review
Source: PLOS Ment Health. 2025 Jun 16;2(6):e0000227. doi: 10.1371/journal.pmen.0000227 (PMC12798439; doi:10.1371/journal.pmen.0000227)
Supplement: S2 Table — (DOCX) [file pmen.0000227.s002.docx]

# **S2 Table. Mental health organisations**

| **No.** | **Organization Name** |
| --- | --- |
| 1 | Anna Freud Centre |
| 2 | Mind UK |
| 3 | Mind Manchester |
| 4 | MindEd |
| 5 | Heads Together |
| 6 | Rethink Mental Illness |
| 7 | Mood Juice |
| 8 | Happy Maps |
| 9 | Brave Online |
| 10 | Stem 4 |
| 11 | Charlie Waller Trust |
| 12 | Hands On Scotland |
| 13 | Help Guide |
| 14 | Place To Be |
| 15 | Young Minds |
| 16 | Bridge The Gap |
| 17 | Kooth |
| 18 | Heads Above The Waves |
| 19 | ACAMH |
| 20 | Anxiety UK |
| 21 | Time To Change |
| 22 | Childline |
| 23 | Edinburgh & Lothians Greenspace Trust |
| 24 | The Mix |
| 25 | Mind Mate |
| 26 | Young Person’s Advisory Service (YPAS) |
