# Supplementary material for: Multi-component interventions combining psychotherapy and physical activity for children and young peoples’ mental health: A scoping review
Source: PLOS Ment Health. 2025 Jun 16;2(6):e0000227. doi: 10.1371/journal.pmen.0000227 (PMC12798439; doi:10.1371/journal.pmen.0000227)
Supplement: S3 Table — (DOCX) [file pmen.0000227.s003.docx]

# **S3 Table. Expert involvement**

| **No.** | **Job Role and Background** | **Years of experience in the field of CYP mental health** | **Involvement Stage** |
| --- | --- | --- | --- |
| 1 | Associate Head. Mental Health and Wellbeing | 7 years | Validating and informing the findings at stage 5 |
| 2 | Senior Lecturer in Counselling & Psychotherapy, and Child and Adolescent Mental Health and Wellbeing | Not stated | Informing the inclusion and exclusion criteria and signposting to relevant studies at stage 2, and validating and informing the findings at stage 5 |
| 3 | Lecturer in Counselling & Psychotherapy.  Person-Centred Experiential Expressive Arts Therapist | Not stated | Signposting to relevant studies at stage 2, and validating and informing the findings at stage 5 |
| 4 | Postdoctoral Researcher | Not stated | Overlooking the search strategy and signposting to relevant studies at stage 2, and validating and informing the findings at stage 5 |
