# Supplementary material for: Multi-component interventions combining psychotherapy and physical activity for children and young peoples’ mental health: A scoping review
Source: PLOS Ment Health. 2025 Jun 16;2(6):e0000227. doi: 10.1371/journal.pmen.0000227 (PMC12798439; doi:10.1371/journal.pmen.0000227)
Supplement: S1 Text — (DOCX) [file pmen.0000227.s004.docx]

# **S1 Text. Search strategy**

CBT OR “cognitive behavio*r therapy” OR REBT OR “rational emotive behavio*r therapy” OR “Behavio*r Therapy” OR “Cognitive Therapy” OR Counsel*ing OR “Talk Therapy” OR BA OR “behavio*ral activation” OR “Mindfulness-Based therapy” OR DMT OR “dance movement therapy” OR DMP OR “dance movement psychotherapy” OR “Psychodynamic Therapy” OR “psychoanalytic* therapy” OR “Humanistic Therapy” OR “Interpersonal Therapy” OR “Dialectical Behavio*r Therapy” OR “Transactional Analysis” OR “Gestalt Therapy” OR “Compassion Focused Therapy” OR “Acceptance Commitment Therapy” OR “Eye Movement Desensitization and Reprocessing” OR “psychotherap*”

AND

“physical activit*” OR “movement*” OR “exercis*”

AND

“Psychological N3 (Wellbeing OR Health OR symptom*) OR Emotional N3 (Health OR wellbeing OR distress OR disorder* OR difficult* OR problem* OR adjustment) OR “mental N3 (health OR wellbeing OR disorder* OR illness*) OR “Resilience” OR “Quality of Life” OR “Anxiety” OR “Depression” OR “Internali*ed disorder*” OR “Externali*ed disorder*” OR “Self-efficacy” OR “Self esteem” OR “Behavior Symptom*” OR “Mental Disorder*” OR “Low Mood” OR “Behavio*r problem*” OR “Affective Disorder*”

AND

Adolescent* OR Teenager* OR Youngster* OR Youth OR Minor* OR Teen* OR Pupil* OR child* OR “school student*”
