# Supplementary material for: Multi-component interventions combining psychotherapy and physical activity for children and young peoples’ mental health: A scoping review
Source: PLOS Ment Health. 2025 Jun 16;2(6):e0000227. doi: 10.1371/journal.pmen.0000227 (PMC12798439; doi:10.1371/journal.pmen.0000227)
Supplement: S2 Text — (DOCX) [file pmen.0000227.s005.docx]

# **S2 Text. Data extraction guidance**

1. Participant Demographics (Gender, Age, Mental Health Status)
2. Article Details (Author, Year, Country, Study Design, Setting)
3. Study Aim
4. Intervention Details (Setting, Delivery, Duration, Session Frequency)
5. Outcome (Measures, Potential Effectiveness, Feasibility, Acceptability, Sustainability)
6. Key Findings
