# Supplementary material for: Multi-component interventions combining psychotherapy and physical activity for children and young peoples’ mental health: A scoping review
Source: PLOS Ment Health. 2025 Jun 16;2(6):e0000227. doi: 10.1371/journal.pmen.0000227 (PMC12798439; doi:10.1371/journal.pmen.0000227)
Supplement: S3 Text — (DOCX) [file pmen.0000227.s006.docx]

# **S3 Text. Primary outcome measures**

CDI: Children’s Depression Inventory; CBCL: Child Behaviour Checklist; TSCC: Trauma Symptom Checklist; BDI: Beck Depression Inventory; BAI: Beck Anxiety Inventory; ADSHS: Adolescent Domain - Specific Hope Scale; MDI: Major Depression Inventory; YQOL-R: Youth Quality of Life-Research Version; QIDS-SR: Quick Inventory of Depression Symptomatology; BDI-II: Beck Depression Inventory – II; MADRS: Montgomery-Åsberg Depression Rating Scale; RSES: Rosenberg Self-Esteem Scale; UCLA PTSD Reaction Index: University of California, Los Angeles Posttraumatic Stress Disorder Reaction Index; SCARED: Screen for Child Anxiety-Related Emotional Disorders; QIDS-A17: Quick inventory of depressive symptomatology, adolescent version; IKL: Inventory for the assessment of quality of life in children and adolescents; KINDL: Self-esteem dimension of the questionnaire for the assessment of health-related quality of life in children; SDQ-Deu: Strengths and Difficulties Questionnaire German translation; BVF-K: Bullying and victimization self-report for children; HAM-D: Hamilton Depression Rating Scale; WHO-5: WHO-Five Well-Being Index; SPPC: Self-perception Profile for Children; GSES: General Self-Efficacy Scale; SOFAS: Social and Occupational Functioning Assessment Scale; SCWS: Stirling Child Well-Being Scale; CYRM: Child and Youth Resilience Measure; SCL-90: Symptom Checklist 90; GHQ-12: Twelve Item General Health Questionnaire; WEMWBS: Warwick Edinburgh Mental Well-Being Scale; AAQ-II: Acceptance and Action Questionnaire-2^nd^ Edition; SDS: Self-rating Depression Scale; SAS: Self-rating Anxiety Scale; PSQI: Pittsburg Sleep Quality Index; YDQ: Young Diagnostic Questionnaire; PANAS: Positive Affect and Negative Affect Scale; PPQ: Positive Psychological Capital Questionnaire; EQ-5D-Y: Quality of Life for Children; CORS: Child Outcome Rating Scale; CSRS: Child Session Rating Scale; SDQ: Strengths and Difficulties Questionnaire; PHQ-A: Patient Health Questionnaire-adapted for Adolescents; BES: Basic Empathy Scale; PROMIS: Patient-Reported Outcomes Measurement Information System; PRSSF: Pediatric Peer Relationships-Short Form 8a; CSES_A: Modified version of the Cultural Self-Efficacy Scale for Adolescents.
