# Supplementary material for: Multi-component interventions combining psychotherapy and physical activity for children and young peoples’ mental health: A scoping review
Source: PLOS Ment Health. 2025 Jun 16;2(6):e0000227. doi: 10.1371/journal.pmen.0000227 (PMC12798439; doi:10.1371/journal.pmen.0000227)
Supplement: S4 Text — (PDF) [file pmen.0000227.s007.pdf]

Friday, November 10, 2023 9:33:54 AM

#	Query	Limiters/Expanders	Last Run Via	Results
S16	S4 AND S8 AND S12 AND S13	Limiters - Published Date: 20130101-20231031; English Language; Language: English<br>Expanders - Apply equivalent subjects<br>Search modes - Find all my search terms	Interface - EBSCOhost Research Databases<br>Search Screen - Advanced Search<br>Database - CINAHL Complete	257
S15	S4 AND S8 AND S12 AND S13	Limiters - Published Date: 20130101-20231231<br>Expanders - Apply equivalent subjects<br>Search modes - Find all my search terms	Interface - EBSCOhost Research Databases<br>Search Screen - Advanced Search<br>Database - CINAHL Complete	266
S14	S4 AND S8 AND S12 AND S13	Expanders - Apply equivalent subjects<br>Search modes - Find all my search terms	Interface - EBSCOhost Research Databases<br>Search Screen - Advanced Search<br>Database - CINAHL Complete	369
S13	TI (Adolescen* OR Teenager* OR Youngster* OR Youth OR Minor* OR Teen* OR Pupil* OR child* OR student*) OR AB (Adolescen* OR Teenager* OR Youngster* OR Youth OR Minor* OR Teen* OR Pupil* OR child* OR student*)	Expanders - Apply equivalent subjects<br>Search modes - Find all my search terms	Interface - EBSCOhost Research Databases<br>Search Screen - Advanced Search<br>Database - CINAHL Complete	988,074
S12	S9 OR S10 OR S11	Expanders - Apply equivalent subjects<br>Search modes - Find all my search terms	Interface - EBSCOhost Research Databases<br>Search Screen - Advanced Search<br>Database - CINAHL Complete	472,357
S11	(MM "Psychological Well-Being")	Expanders - Apply equivalent subjects<br>Search modes - Find all my search terms	Interface - EBSCOhost Research Databases<br>Search Screen - Advanced Search<br>Database - CINAHL Complete	17,473
S10	(MM "Mental Health")	Expanders - Apply equivalent subjects<br>Search modes - Find all my search terms	Interface - EBSCOhost Research Databases<br>Search Screen - Advanced Search<br>Database - CINAHL Complete	32,246
S9	TI (“Psychological N3 (Wellbeing OR Health OR symptom*) OR Emotional N3 (Health OR wellbeing OR distress OR disorder* OR difficult* OR problem* OR adjustment) OR “mental N3 (health OR wellbeing OR disorder* OR illness*) OR Resilience OR Quality of Life OR Anxiety OR Depression OR “Internal* disorder*” OR “External* disorder*” OR “Self efficacy” OR “Self esteem” OR “Behavio#r* Symptom*” OR “Mental Disorder*” OR “Low Mood” OR “Behavio#r* problem*” OR “Affective Disorder*”) OR AB (“Psychological N3 (Wellbeing OR Health OR symptom*) OR Emotional N3 (Health OR wellbeing OR distress OR disorder* OR difficult* OR problem* OR adjustment) OR “mental N3 (health OR wellbeing OR disorder* OR illness*) OR Resilience OR Quality of Life OR Anxiety OR Depression OR “Internal* disorder*” OR “External* disorder*” OR “Self efficacy” OR “Self esteem” OR “Behavio#r* Symptom*” OR “Mental Disorder*” OR “Low Mood” OR “Behavio#r* problem*” OR “Affective Disorder*”)	Expanders - Apply equivalent subjects<br>Search modes - Find all my search terms	Interface - EBSCOhost Research Databases<br>Search Screen - Advanced Search<br>Database - CINAHL Complete	438,122

S8	S5 OR S6 OR S7	Expanders - Apply equivalent subjects<br>Search modes - Find all my search terms	Interface - EBSCOhost Research Databases<br>Search Screen - Advanced Search<br>Database - CINAHL Complete	286,711
S7	(MM "Exercise")	Expanders - Apply equivalent subjects<br>Search modes - Find all my search terms	Interface - EBSCOhost Research Databases<br>Search Screen - Advanced Search<br>Database - CINAHL Complete	33,444
S6	(MM "Physical Activity")	Expanders - Apply equivalent subjects<br>Search modes - Find all my search terms	Interface - EBSCOhost Research Databases<br>Search Screen - Advanced Search<br>Database - CINAHL Complete	29,700
S5	TI (“physical activit*” OR “movement OR exercis*) OR AB (“physical activit*” OR “movement OR exercis*)	Expanders - Apply equivalent subjects<br>Search modes - Find all my search terms	Interface - EBSCOhost Research Databases<br>Search Screen - Advanced Search<br>Database - CINAHL Complete	275,924
S4	S1 OR S2 OR S3	Expanders - Apply equivalent subjects<br>Search modes - Find all my search terms	Interface - EBSCOhost Research Databases<br>Search Screen - Advanced Search<br>Database - CINAHL Complete	109,341
S3	(MM "Mental Health Counseling")	Expanders - Apply equivalent subjects<br>Search modes - Find all my search terms	Interface - EBSCOhost Research Databases<br>Search Screen - Advanced Search<br>Database - CINAHL Complete	41
S2	(MM "Psychotherapy")	Expanders - Apply equivalent subjects<br>Search modes - Find all my search terms	Interface - EBSCOhost Research Databases<br>Search Screen - Advanced Search<br>Database - CINAHL Complete	14,379
S1	TI (CBT OR “cognitive behavio#r*” OR REBT OR “rational emotive behavio#r* therap*” OR “Behavio#r* Therap*” OR “Cognitive Therap*” OR Counsel*ing OR “Talk Therap*” OR BA OR “behavio#r* activation” OR “Mindfulness Based therap*” OR DMT OR “dance movement therap*” OR DMP OR “dance movement psychotherap*” OR “Psychodynamic Therap*” OR “psychoanalytic therap*” OR “Humanistic Therap*” OR “Interpersonal Therap*” OR “Dialectic* Behavio#r* Therap*” OR “Transactional Analysis” OR “Gestalt Therap*” OR “Compassion Focused Therap*” OR “Acceptance Commitment Therap*” OR “Eye Movement Desensitization and Reprocessing” OR psychotherap* OR “psychological therap*”) OR AB (CBT OR “cognitive behavio#r*” OR REBT OR “rational emotive behavio#r* therap*” OR “Behavio#r* Therap*” OR “Cognitive Therap*” OR Counsel*ing OR “Talk Therap*” OR BA OR “behavio#r* activation” OR “Mindfulness Based therap*” OR DMT OR “dance movement therap*” OR DMP OR “dance movement psychotherap*” OR “Psychodynamic Therap*” OR “psychoanalytic therap*” OR “Humanistic Therap*” OR “Interpersonal Therap*” OR “Dialectic* Behavio#r* Therap*” OR “Transactional Analysis” OR “Gestalt Therap*” OR “Compassion Focused Therap*” OR “Acceptance Commitment Therap*” OR “Eye Movement Desensitization and Reprocessing” OR psychotherap* OR “psychological therap*”)	Expanders - Apply equivalent subjects<br>Search modes - Find all my search terms	Interface - EBSCOhost Research Databases<br>Search Screen - Advanced Search<br>Database - CINAHL Complete	102,199

Tue, April 22, 2025 05:13:06 pm

#	Query	Limiters/Expanders	Last Run Via	Results
S16	S4 AND S8 AND S12 AND S13	Limiters - Publication Date: 20231001-20250431<br>Expanders - Apply equivalent subjects<br>Narrow by Language: - english<br>Search modes - Find all my search terms	Interface - EBSCOhost Research Databases<br>Search Screen - Advanced Search<br>Database - CINAHL Complete	53
S15	S4 AND S8 AND S12 AND S13	Limiters - Publication Date: 20231001-20250431<br>Expanders - Apply equivalent subjects<br>Search modes - Find all my search terms	Interface - EBSCOhost Research Databases<br>Search Screen - Advanced Search<br>Database - CINAHL Complete	54
S14	S4 AND S8 AND S12 AND S13	Expanders - Apply equivalent subjects<br>Search modes - Find all my search terms	Interface - EBSCOhost Research Databases<br>Search Screen - Advanced Search<br>Database - CINAHL Complete	445
S13	TI (Adolescen* OR Teenager* OR Youngster* OR Youth OR Minor* OR Teen* OR Pupil* OR child* OR student*) OR AB (Adolescen* OR Teenager* OR Youngster* OR Youth OR Minor* OR Teen* OR Pupil* OR child* OR student*)	Expanders - Apply equivalent subjects<br>Search modes - Find all my search terms	Interface - EBSCOhost Research Databases<br>Search Screen - Advanced Search<br>Database - CINAHL Complete	1,028,164

S12	S9 OR S10 OR S11	Expanders - Apply equivalent subjects<br>Search modes - Find all my search terms	Interface - EBSCOhost Research Databases<br>Search Screen - Advanced Search<br>Database - CINAHL Complete	545,147
S11	(MH "Psychological Well-Being")	Expanders - Apply equivalent subjects<br>Search modes - Find all my search terms	Interface - EBSCOhost Research Databases<br>Search Screen - Advanced Search<br>Database - CINAHL Complete	48,873
S10	(MH "Mental Health")	Expanders - Apply equivalent subjects<br>Search modes - Find all my search terms	Interface - EBSCOhost Research Databases<br>Search Screen - Advanced Search<br>Database - CINAHL Complete	69,970
S9	TI (“Psychological N3 (Wellbeing OR Health OR symptom*) OR Emotional N3 (Health OR wellbeing OR distress OR disorder* OR difficult* OR problem* OR adjustment) OR “mental N3 (health OR wellbeing OR disorder* OR illness*) OR Resilience OR Quality of Life OR Anxiety OR Depression OR “Internal* disorder*” OR “External* disorder*” OR “Self efficacy” OR “Self esteem” OR “Behavio#r* Symptom*” OR “Mental Disorder*” OR “Low Mood” OR “Behavio#r* problem*” OR “Affective Disorder*”) OR AB (“Psychological N3 (Wellbeing OR Health OR symptom*) OR Emotional N3 (Health OR wellbeing OR distress OR disorder* OR difficult* OR problem* OR adjustment) OR “mental N3 (health OR wellbeing OR disorder*	Expanders - Apply equivalent subjects<br>Search modes - Find all my search terms	Interface - EBSCOhost Research Databases<br>Search Screen - Advanced Search<br>Database - CINAHL Complete	464,883

	OR illness*) OR Resilience OR Quality of Life OR Anxiety OR Depression OR “Internal* disorder*” OR “External* disorder*” OR “Self efficacy” OR “Self esteem” OR “Behavio#r* Symptom*” OR “Mental Disorder*” OR “Low Mood” OR “Behavio#r* problem*” OR “Affective Disorder*”)			
S8	S5 OR S6 OR S7	Expanders - Apply equivalent subjects<br>Search modes - Find all my search terms	Interface - EBSCOhost Research Databases<br>Search Screen - Advanced Search<br>Database - CINAHL Complete	309,310
S7	(MH "Exercise")	Expanders - Apply equivalent subjects<br>Search modes - Find all my search terms	Interface - EBSCOhost Research Databases<br>Search Screen - Advanced Search<br>Database - CINAHL Complete	62,175
S6	(MM "Physical Activity")	Expanders - Apply equivalent subjects<br>Search modes - Find all my search terms	Interface - EBSCOhost Research Databases<br>Search Screen - Advanced Search<br>Database - CINAHL Complete	32,707
S5	TI (“physical activit*” OR “movement OR exercis*) OR AB (“physical activit*” OR “movement OR exercis*)	Expanders - Apply equivalent subjects<br>Search modes - Find all my search terms	Interface - EBSCOhost Research Databases<br>Search Screen - Advanced Search<br>Database - CINAHL Complete	282,005
S4	S1 OR S2 OR S3	Expanders - Apply equivalent subjects<br>Search modes - Find all my search terms	Interface - EBSCOhost Research Databases<br>Search Screen - Advanced Search<br>Database - CINAHL Complete	119,134
S3	(MM "Mental Health Counseling")	Expanders - Apply equivalent subjects<br>Search modes - Find all my search terms	Interface - EBSCOhost Research Databases<br>Search Screen - Advanced Search<br>Database - CINAHL Complete	71

S2	(MH "Psychotherapy")	Expanders - Apply equivalent subjects<br>Search modes - Find all my search terms	Interface - EBSCOhost Research Databases<br>Search Screen - Advanced Search<br>Database - CINAHL Complete	25,839
S1	TI (CBT OR “cognitive behavio#r*” OR REBT OR “rational emotive behavio#r* therap*” OR “Behavio#r* Therap*” OR “Cognitive Therap*” OR Counsel*ing OR “Talk Therap*” OR BA OR “behavio#r* activation” OR “Mindfulness Based therap*” OR DMT OR “dance movement therap*” OR DMP OR “dance movement psychotherap*” OR “Psychodynamic Therap*” OR “psychoanalytic therap*” OR “Humanistic Therap*” OR “Interpersonal Therap*” OR “Dialectic* Behavio#r* Therap*” OR “Transactional Analysis” OR “Gestalt Therap*” OR “Compassion Focused Therap*” OR “Acceptance Commitment Therap*” OR “Eye Movement Desensitization and Reprocessing” OR psychotherap* OR “psychological therap*”) OR AB (CBT OR “cognitive behavio#r*” OR REBT OR “rational emotive behavio#r* therap*” OR “Behavio#r* Therap*” OR “Cognitive Therap*” OR Counsel*ing OR “Talk Therap*” OR BA OR “behavio#r* activation” OR “Mindfulness Based therap*” OR DMT	Expanders - Apply equivalent subjects<br>Search modes - Find all my search terms	Interface - EBSCOhost Research Databases<br>Search Screen - Advanced Search<br>Database - CINAHL Complete	103,654

OR “dance movement therap*” OR DMP OR “dance movement psychotherap*” OR “Psychodynamic Therap*” OR “psychoanalytic therap*” OR “Humanistic Therap*” OR “Interpersonal Therap*” OR “Dialectic* Behavio#r* Therap*” OR “Transactional Analysis” OR “Gestalt Therap*” OR “Compassion Focused Therap*” OR “Acceptance Commitment Therap*” OR “Eye Movement Desensitization and Reprocessing” OR psychotherap* OR “psychological therap*”)
